# Primary adenoid cystic carcinoma of the breast: case report and review of the literature

**DOI:** 10.1186/1477-7800-3-17

**Published:** 2006-06-30

**Authors:** Alaa A Muslimani, Manmeet S Ahluwalia, Christopher T Clark, Hamed A Daw

**Affiliations:** 1 Department of Internal Medicine, Fairview Hospital, 18101, Lorain Avenue, Cleveland, OH, USA; 2 Department of Pathology, Fairview Hospital, 18101, Lorain Avenue, Cleveland, OH, USA; 3 Cleveland Clinic Health System and the Cleveland Clinic Cancer Center, Moll Pavilion, 18200 Lorain Avenue, Cleveland, OH, USA

## Abstract

Adenoid cystic carcinoma (ACC) of the breast is a rare neoplasm accounting for 0.1% of all breast carcinomas, and presenting most commonly as a painful breast mass. In contrast to the aggressive nature of ACC at other sites, ACC of the breast has a favorable prognosis, lymph node involvement or distant metastases seldom occur. Treatment is basically of simple mastectomy. Chemotherapy, radiation and hormonal treatment have been infrequently used and evaluated. We report a case of ACC of the breast managed with mastectomy and review the literature.

## Background

Adenoid cystic carcinoma (ACC) of the breast is a rare breast cancer variant and optimal management is unclear. Salivary gland tumors having the same histological pattern as ACC of the breast were first termed "cylindroma" by Billroth [2]. Although ACC of the breast can occur between 30 and 90 years of age, it is more common in women in the fifth and sixth decade of life [3]. Most patients present with a dominant breast mass tender to palpation [4,7]. Histologically, ACC has a unique distinctive biphasic pattern that consists of true laminate and pseudo- cystic spaces, true glands are lined by epithelial cells and pseudocysts are lined by myoepithelial cells.

Cytologically, the tumor shows a typical pattern; globules of mucous surrounded by epithelial cells with little cytoplasm and small hyperchromatic nuclei.

It reportedly has a better prognosis than most forms of breast cancer and the incidence of axillary lymph node metastases is lower [8]. Distant metastases are uncommon, however when they occur they tend to do so without prior lymph node involvement. It is generally estrogen receptor (ER) and progesterone receptor (PR) negative.

## Case presentation

A seventy-one-year-old single female presented with a small tender lump in the inferior quadrant of her right breast. Initial mammography and ultrasonography of the breast were inconclusive. Her past medical history was significant for vaginal hysterectomy at 45 years of age and back surgery at 68 years of age for degenerative spine disease. Family history was negative for breast cancer but her father had colon cancer. She was a non-smoker and did not consume alcohol. Three weeks later examination showed no change in size but increased tenderness. However, no erythema, ecchymosis, skin ulceration or dimpling was seen. An excisional biopsy showed ACC. She subsequently underwent a modified radical mastectomy with axillary node dissection.

## Pathology

The tumor measured 1.2 cm in maximum diameter. The margins were free of carcinoma. Both the nuclear grade of the lesion and the Bloom Richardson grade was 1. No involvement of the nipple or the skin was present. Lymph nodes were negative for tumor metastasis. The tumor was staged, as T1N0M0, and was ER and PR negative. It was DNA diploid with a low S-phase fraction. The Her-cep test™ was negative.

## Discussion

ACC of the breast is a rare neoplasm accounting for 0.1% of all breast carcinomas [1]. It is of special interest because of its favorable prognosis and distinctive histological appearance [4]. Although ACC occurs predominantly in females, male patients have been described in the literature [9,10]. ACC most frequently presents as a tender breast mass, often in the subareolar area. Prognosis is more favorable in ACC as compared to other types of breast cancer as lymph node involvement and distant metastasis are uncommon. In contrast to the aggressive nature of the salivary gland tumors, ACC of the breast has a favorable prognosis. The reason for this difference is unexplained. As seen in our patient, most patients with ACC have a mass when first diagnosed [12] and pain is often the most common symptom [4,6]. Patients have been reportedly monitored for more than a year with just the symptom of breast pain before a mass or a radiographic abnormality is detected [12]. In this case, the patient complained of pain although the initial mammography and the USG were inconclusive. Pain is also seen in ACC of the salivary gland where the pain and the tenderness are attributed to perineural invasion by the tumor. However in our patient such invasion was absent. This has been described in other series as well [12]. Although ACC of the breast is most commonly subareolar in location, nipple discharge is an uncommon symptom in ACC [13], and was not seen in our patient. Most ACC are well circumscribed and firm. The diagnosis can be made on fine needle aspiration cytology [14,15]. The smears are highly cellular and contain extracellular spheres of metachromatic material surrounded by uniform cells with scant cytoplasm. The tumor is mostly well circumscribed in appearance and generally ranges from 1 to 5 cm in size [5]. Ro et al. suggested that ACC of the breast can be graded on the proportion of solid growth of the tumor and this was correlated with prognosis [16]. Grades proposed were 1 (no solid element); grade 2 (< 30 % solid element); grade 3 (>30% solid element). The proposed treatment was local excision for grade 1 tumors, simple mastectomy for grade 2 tumors and mastectomy with axillary node dissection for grade 3 tumors. Although calcification may develop in these tumors, only infrequently are they detected by mammography. In our patient the initial mammogram was inconclusive as screening mammography often misses the tumor. In another series only 4 of 22 patients detected radiologically [12]. Axillary lymph node metastases are rare in patients with ACC. Arpino et al. noted lymph node metastases in only 4 of the 182 cases collected from the literature [3]. Distant metastases are uncommon with only 14 cases having been reported, but when they occur they tend to do so without prior lymph node involvement. Hence a routine axillary LN dissection is not recommended [3].

ACC is generally ER negative; in various series the ER was described as positive in 0.7% to 28 % of the cases [12,17]. PR was positive in 3 of the 13 evaluated patients [12]. The best surgical treatment for ACC of the breast has not been established. Local excision is followed by unacceptably high rates of recurrence [8]. The studies showed that ACC has low proliferation activity which may explain the low recurrence rate. Based on these findings simple mastectomy or lumpectomy followed by radiation treatment is thought to have a chance to achieve adequate local control of nearly all tumors [3-12].

Despite its infrequent use, our patient had a modified radical mastectomy. Neither adjuvant chemotherapy nor hormonal manipulation has been studied in patients with ACC of the breast. No conclusions have been drawn regarding radiation and chemotherapy. Since it is a rare neoplasm clinical trials comparing treatment options for ACC are needed to define the optimal treatment.

**Table 1:** Adenoid cystic carcinoma of the breast: At a glance.

*Incidence:*
• 0.1% of all breast cancers.
*Age at presentation:*
• 30 to 90 (mostly in 5th and 6th decade of life).
*Most common presentation:*
• Painful breast mass.
*Mammography (role):*
• Limited as compared to other breast cancers (may miss the tumor).
*Lymph node involvement:*
• Uncommon.
*Metastases:*
• Rare (can occur without LN involvement).
*Most common site of metastases:*
• Lung.

***Management Options:***
*Surgery*
• Simple Mastectomy (most widely performed).
• Lumpectomy followed by radiation (alternative option).
• Modified radical mastectomy (falling out of favor, considered too radical).
*Radiation:*
• Insufficiently evaluated (follows lumpectomy).
*Chemotherapy:*
• Not studied.
*Hormonal manipulation:*
• Of limited value as most cases are ER & PR negative.

**Figure 1a:**
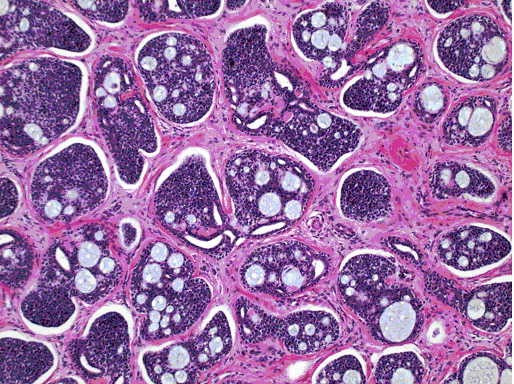
100× magnification of the breast mass showing islands of cells with a characteristic cribriform pattern with a fibrous desmoplastic background. H & E stain.

**Figure 1b:**
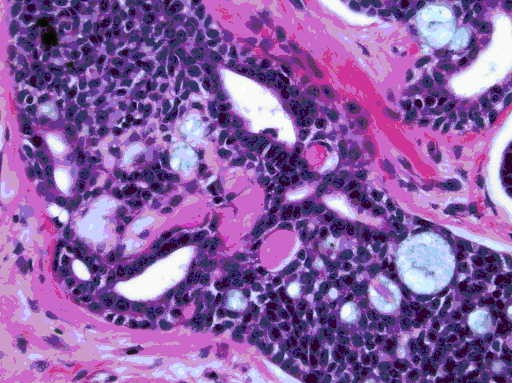
400× magnification view showing the cribriform architecture with some lumen containing bluish material. Also seen are the characteristic balls of eosinophilic material. H & E stain.
